# Predictive Modeling of Indoor Environmental Parameters for Assessing Comfort Conditions in a Kindergarten Setting

**DOI:** 10.3390/toxics11080709

**Published:** 2023-08-17

**Authors:** Radostin Mitkov, Dessislava Petrova-Antonova, Petar O. Hristov

**Affiliations:** 1 GATE Institute, Sofia University “St. Kliment Ohridski”, 1113 Sofia, Bulgaria; dessislava.petrova@gate-ai.eu (D.P.-A.); petar.hristov@gate-ai.eu (P.O.H.); 2 Faculty of Mathematics and Informatics, Sofia University “St. Kliment Ohridski”, 1164 Sofia, Bulgaria; 3 Institute for Risk and Uncertainty, School of Engineering, University of Liverpool, Liverpool L69 7ZF, UK

**Keywords:** predictive modeling, machine learning, indoor environment, comfort conditions, air quality

## Abstract

People tend to spend the majority of their time indoors. Indoor air properties can significantly affect humans’ comfort, health, and productivity. This study utilizes measurement data of indoor conditions in a kindergarten in Sofia, Bulgaria. Autoregressive integrated moving average (ARIMA) and long short-term memory (LSTM) recurrent neural network (RNN) models were developed to predict CO${}_{2}$ levels in the educational facility over the next hour based on 2.5 h of past data and allow for near real-time decision-making. The better-performing model, LSTM, is also used for temperature and relative humidity forecasting. Global comfort is then estimated based on threshold values for temperature, humidity, and CO${}_{2}$. The predicted $R^{2}$ values ranged between 0.938 and 0.981 for the three parameters, while the prediction of global comfort conditions achieved a 91/100 accuracy.

## 1. Introduction

Most people nowadays tend to spend 90% of their time indoors—at home, at work, in school, or other enclosed environments [1,2]. Numerous studies have linked poor indoor conditions with symptoms such as eye, nose, and throat irritation; headaches; fatigue; skin dryness; and asthma-like symptoms [3,4,5,6]. These symptoms are most commonly observed in indoor spaces with a high number of occupants [7] and are connected to a condition named “sick building syndrome” (SDS) [8]. Even short-term exposure to inadequate indoor conditions could cause harm to more sensitive groups such as children or the elderly or people with other health conditions such as cardiovascular or respiratory diseases [9,10]. In fact, young children are at even higher risk due to their 2–3 times higher volume of inhaled air with respect to their body weight compared to adults [11].

The quality of the indoor environment in educational facilities is often determined by different factors such as air pollution, temperature, relative humidity, ventilation rate, concentration of volatile organic compounds, etc. Particulate matter (PM${}_{10}$ and PM${}_{2.5}$), for instance, has generally been associated in several studies with increased mortality rate, and respiratory and cardiovascular conditions, especially among children [12,13]. The Indoor Air Hygiene Institute states that PM${}_{2.5}$ levels above 35 μg/m${}^{3}$ during a 24 h period are considered unhealthy [14]. Carbon dioxide (CO${}_{2}$) concentrations above 1000 ppm have been proven to contribute to headaches, decreased cognitive performance, dry cough, and respiratory problems in young children [15,16,17]. Low ventilation rates, high temperatures, and low relative humidity also have a negative influence on the children’s overall learning performance [18,19]. Therefore, the environmental conditions in kindergartens and schools play a vital role in the children’s learning efficiency, short- and long-term health, and well-being.

Monitoring different indoor parameters in real time is essential to ensuring a healthy environment. The availability and affordability of computational resources over the past few decades have enabled the utilization of various models, including artificial intelligence (AI) and machine learning (ML) models, for the prediction of future environmental conditions [20]. The most commonly used models in such forecasting studies are regression models, support vector machines, artificial neural networks (ANN), decision trees, random forests, and generalized boosting models [21].

The aim of this study is to comprehensively assess the indoor environmental conditions and present a short-term prediction of the comfort conditions inside a kindergarten room in Sofia, Bulgaria using a recurrent neural network (RNN). While certain parameters such as particulate matter, temperature, and relative humidity have received considerable attention in previous research [20], the inclusion of CO${}_{2}$ as a critical parameter is of significant importance. Monitoring, control, and prediction of CO${}_{2}$ alongside other parameters provides a holistic understanding of the indoor environment. The rest of this article is structured as follows: Section 2 describes the materials and methods of this study. Section 3 then demonstrates the results and discussions. Finally, Section 4 presents the conclusion and future works.

## 2. Materials and Methods

### 2.1. Site Description

The current study employs data from a kindergarten room located in a residential area in Sofia, Bulgaria. The selected indoor space is located on the ground level and is naturally ventilated. The room is occupied from Monday to Friday between 7:00 a.m. and 5:30 p.m. except holiday periods.

### 2.2. Data Collection and Instrumentation

The measuring equipment is placed on a wall opposite the windows. It consists of temperature and relative humidity sensor (https://www.bosch-sensortec.com/media/boschsensortec/downloads/datasheets/bst-bme280-ds002.pdf (accessed on 12 July 2023)), a fine particulate matter sensor (https://eu.mouser.com/datasheet/2/187/HWSC_S_A0012942921_1-3073234.pdf (accessed on 12 July 2023)), and a CO${}_{2}$ sensor (https://f.hubspotusercontent40.net/hubfs/9035299/Documents/AAS-920-634G-Telaire-T6713-Series-011321-web.pdf (accessed on 12 July 2023)). The measurement ranges are, respectively, [−40, 85] ${}^{{}^{\circ}}$C, [0, 100]%, [0, 1000] μg/m${}^{3}$, and [0, 5000] ppm. The measurement ranges adequately cover the air property values expected to be observed in the current study. The fine particulate matter sensor measures PM${}_{2.5}$ and provides estimates for PM${}_{1.0}$, PM${}_{4}$, and PM${}_{10}$ based on the PM${}_{2.5}$ values. A sound alarm signals the teachers that the CO${}_{2}$ levels have crossed the acceptable threshold of 1000 ppm and urges them to open the windows.

The sampling campaign took place between August 2021 and October 2022 with measurements recorded at irregular intervals.

### 2.3. Data Preparation and Analysis

The whole data set consists of 97,637 records, which are resampled into 15 min interval measurements using weighted mean. The resulting data set consists of 43,872 observations. Around a quarter of those observations, however, have missing values. Forward filling is used in cases of up to two consecutive missing values while longer periods of missing data are removed. The more significant such intervals are (1) 14 September–6 October 2021, (2) 29 April–9 June 2022, (3) 16 June–27 July 2022, (4) 5 August–11 August 2022, and (5) 5 October–13 October 2022. Finally, this results in a data set with 31,524 observations whose descriptive statistics are presented in Table 1.

**Figure 1:**
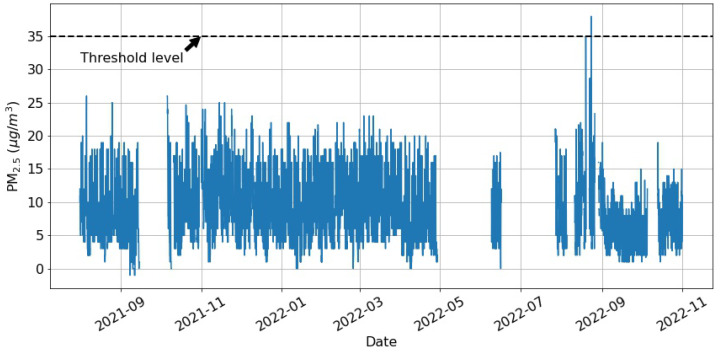
PM${}_{2.5}$ measurements over the whole recorded period. The horizontal line represents the threshold level of 35 μg/m${}^{3}$.

It can be seen that the maximum values of both the CO${}_{2}$ and PM${}_{2.5}$ levels exceed the recommended thresholds. Further investigation reveals that PM${}_{2.5}$ exceeds the limit of 35 μg/m${}^{3}$ only five times during the recorded period of over a year, as observed in Figure 1.

**Figure 2:**
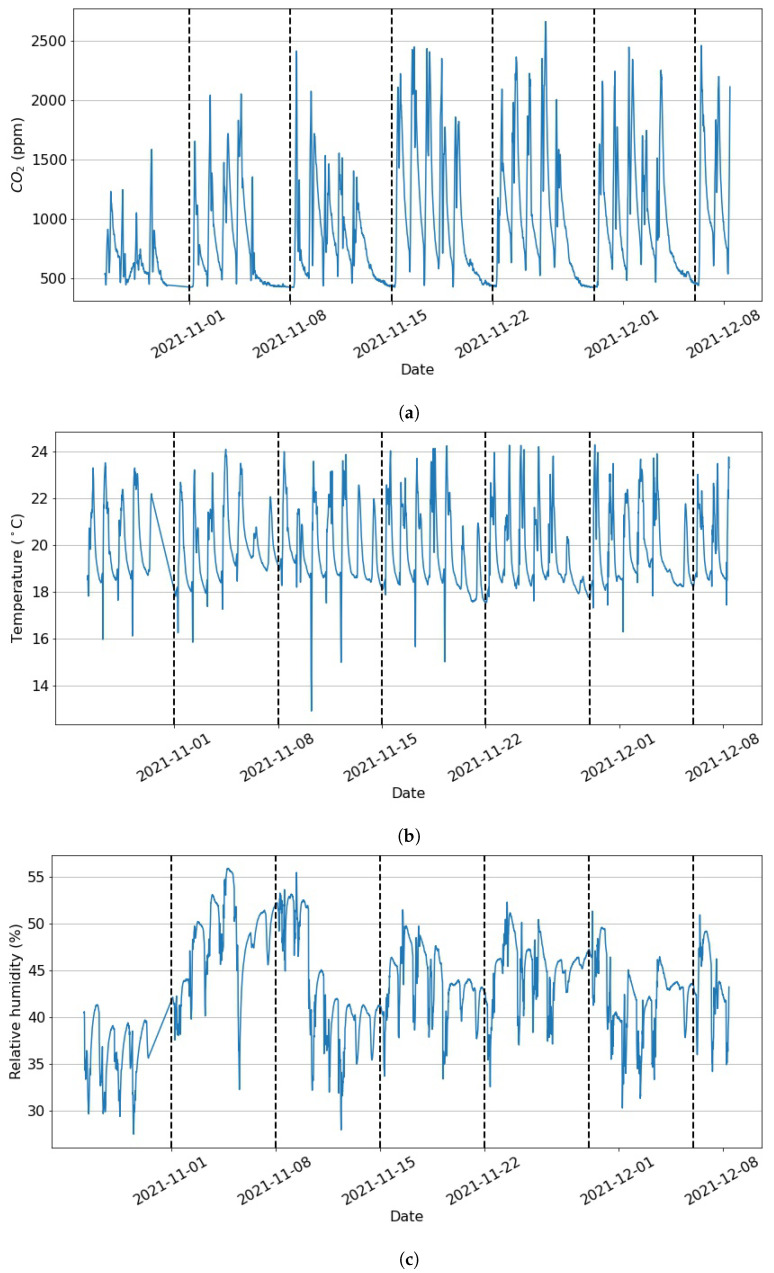
Example (**a**) CO${}_{2}$, (**b**) temperature, and (**c**) relative humidity data. The vertical lines present the end of a week and the beginning of a new one.

Potential reasons for this could be low levels of PM${}_{2.5}$ in the outdoor air in the vicinity of the kindergarten and regular cleaning routines in the facility. On the other hand, during the days when the kindergarten room is occupied (between 7:00 a.m. and 5:30 p.m.), the mean concentration of CO${}_{2}$ is greater than the allowed concentration of 1000 ppm. Thus, this study will focus on predicting CO${}_{2}$ levels and comfort conditions, which pose a much greater threat to the children in this kindergarten room. Figure 2 shows an excerpt of the data representing the CO${}_{2}$, temperature, and relative humidity measurements over a period of around 40 days. It is observed that the CO${}_{2}$ levels exceed the threshold level of 1000 ppm on a daily basis. In fact, further analysis shows that unhealthy levels of CO${}_{2}$ are present more than 15% of the time and more than 23% of the time during the days between 7:00 a.m. and 5:30 p.m. As seen in Figure 2, a cyclical pattern is observed where the measured parameters exhibit peaks during the days and significantly lower values over the nights. In addition, the CO${}_{2}$ plot (Figure 2a) reveals a notable drop in concentrations during the weekends. Thus, the time of the day and the type of day (weekdays or weekends) are considered as additional features in the presented study.

In order to ensure that the models are trained on data from all the year round, the data set is randomly split into training, validation, and testing in an 80:10:10 ratio, which is a commonly reported split in literature [22,23,24]. Because the various features display different ranges and orders of magnitude, all data are re-scaled into the range between 0 and 1 using min–max normalization so that they can be easily interpreted on the same scale. Additional data from 16 May to 15 June 2023 were acquired at a later stage and are used only as a testing data set.

### 2.4. ARIMA

The autoregressive integrated moving average (ARIMA) model combines the differenced autoregressive model with the moving average model to perform time series forecasting [25]. The autoregressive (AR) model expresses the observation at the current time, $\gamma_{t}$, as a linear combination of observations at *p* previous times. Thus, the model has the form:

(1) $$\gamma_{t}=\alpha_{0}+\alpha_{1}\gamma_{t-1}+\alpha_{2}\gamma_{t-2}+...+\alpha_{p}\gamma_{t-p}+e$$

where γ are the observations, $\alpha_{0}$ is a constant, $\alpha_{i}$ are the regression coefficients, and *e* is an error term, usually a zero-mean Gaussian noise. The number *p* is selected with the help of a partial autocorrelation function (PACF) [26].

The differenced model (I) represents the change between consecutive data points in a series and is used to convert a non-stationary time series into a stationary one. The presence of non-stationarity in the time series, and thereby the need for differencing, can be determined through the augmented Dickey–Fuller (ADF) test [27]. First-order differencing can be expressed as:

(2) $$\gamma_{t}^{{}^{\prime }}=\gamma_{t}-\gamma_{t-1}.$$

In a moving average (MA) model the forecast error is linearly dependent on past respective errors. It can be expressed as:

(3) $$\gamma_{t}=I+e_{t}+\theta_{1}e_{t-1}+\theta_{2}e_{t-2}+...+\theta_{q}e_{t-q}$$

where $e_{i}$ is the forecast error of data point *i*, θ are the regression coefficients, *q* is the order of the moving average, and *I* is a constant. The order of *q* is obtained based on the autocorrelation function (ACF) [26]. Combining all three components, the ARIMA model can be expressed as:

(4) $$\gamma_{t}^{{}^{\prime }}=I+\alpha_{1}\gamma_{t-1}^{{}^{\prime }}+\alpha_{2}\gamma_{t-2}^{{}^{\prime }}+...+\alpha_{p}\gamma_{t-p}^{{}^{\prime }}+e_{t}+\theta_{1}e_{t-1}+\theta_{2}e_{t-2}+\theta_{q}e_{t-q}.$$

### 2.5. Long Short-Term Memory (LSTM) Recurrent Neural Network (RNN)

A long short-term memory (LSTM) network is a variation of a recurrent neural network (RNN) that handles the problem of storing long-term information [28]. In the conventional RNN, the backpropagated errors tend to either vanish or increase exponentially. An LSTM architecture consists of three gates—input gate, forget gate, and output gate—which control the flow of information. The input gate controls the input data that flows into the cell state, which stores the long-term memory of past events. The forget gate determines whether information from the previous time step is kept or forgotten. Finally, the output gate is used to decide the value of the next hidden state. The gates’ outputs can be described as follows [29]:

(5) $$i_{t}=\sigma \left(W^{\left(i\right)}x_{t}+U^{\left(i\right)}h_{t-1}+b^{\left(i\right)}\right)$$

(6) $$f_{t}=\sigma \left(W^{\left(f\right)}x_{t}+U^{\left(f\right)}h_{t-1}+b^{\left(f\right)}\right)$$

(7) $$o_{t}=\sigma \left(W^{\left(o\right)}x_{t}+U^{\left(o\right)}h_{t-1}+b^{\left(o\right)}\right)$$

(8) $$g_{t}={\tilde{C}}_{t}=\tanh\left(W^{\left(g\right)}x_{t}+U^{\left(g\right)}h_{t-1}+b^{\left(g\right)}\right)$$

where $i_{t}$, $f_{t}$, and $o_{t}$ are, respectively, input, forget, and output gates; σ is the gate activation function; ${\tilde{C}}_{t}$ is a candidate to a cell memory; $W^{\left(\cdot \right)}$ and $U^{\left(\cdot \right)}$ are weight matrices; $x_{t}$ is the input data; $h_{t-1}$ is the output of previous cells; and $b^{\left(\cdot \right)}$ are bias terms.

Then, a new memory state value, $C_{t}$, and a cell output, $h_{t}$, are calculated using the following equations:

(9) $$C_{t}=g_{t}i_{t}+f_{t}C_{t-1}$$

(10) $$h_{t}=o_{t}\tanh\left(C_{t}\right).$$

### 2.6. Comfort Conditions

The comfort conditions refer to the measurements of temperature, relative humidity, and CO${}_{2}$. The threshold levels, L, for each parameter are defined in accordance with national and international regulations, as summarized in Table 2. The optimal conditions for temperature and relative humidity are set to adhere to the guidelines provided by the Bulgarian regulation for kindergartens [30], where it is recommended to maintain the temperature between $L1=18{\;}^{{}^{\circ}}$C and $L2=21{\;}^{{}^{\circ}}$C and the relative humidity between L1=30% and L2=60%. As previously stated, CO${}_{2}$ levels above 1000 ppm are considered unhealthy. Levels between L1=600 ppm and L2=1000 ppm are characterized as acceptable, while CO${}_{2}$ concentration below 600 ppm is most desirable and constitutes a healthy environment [31]. Using these threshold levels for the three parameters, 27 different indoor comfort conditions can be defined, as described in Table 3, with 1 signifying the greatest comfort and 27–the least comfort. It is important to note that unlike the other parameters under investigation, which exhibit well-defined threshold ranges, PM${}_{2.5}$ only has a single critical value. This led us to focus our detailed analysis on temperature, relative humidity, and CO${}_{2}$ levels. Moreover, the infrequent occurrence of elevated PM${}_{2.5}$ levels indicates a limited impact on the overall indoor environmental quality and occupant comfort conditions.

## 3. Results and Discussion

### 3.1. CO${}_{2}$ Concentrations Prediction—ARIMA vs. LSTM

**Figure 3:**
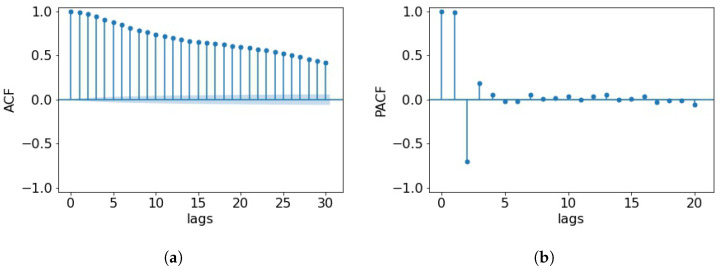
(**a**) ACF and (**b**) PACF plots of the whole data set. Blue shading in (**a**) depicts the significance region.

For the prediction of CO${}_{2}$ concentrations in the kindergarten room, ARIMA and LSTM models are used. The parameters for AR (*p*), the lag difference (*d*), and MA (*q*) are chosen based on ACF and PACF plots, shown in Figure 3, and the ADF test. The ADF test suggests that the time series is already stationary, which means that no differencing is needed. Despite the fact that all investigated lags up to p=30 are indicated as significant in the ACF plot in Figure 3a, the value of *p* is selected to be 10, which represents a trade-off between choosing a sufficient number of past measurements and computational cost. In addition, the behavior of the PACF (Figure 3b) indicates that the degree to which the current time point is directly influenced by events more than 10 lags apart is insignificant. Finally, based on the slow change of the ACF and the long autoregressive behavior, the value of *q* is chosen as zero. Thus the final predictor reduces to a pure AR model.

The architecture of the RNN used throughout this study is the same for all three predicted variables. It consists of four layers, two LSTM and two fully connected hidden layers with respective outputs and parameters shown in Table 4. The RNN utilizes the Adam optimizer with $\beta_{1}=0.9$, $\beta_{2}=0.999$, and $\epsilon =10^{-8}$, as recommended in [32]. In addition, a learning rate schedule is defined using exponential decay with an initial learning rate of 0.05, 400 decay steps, and a decay rate of 0.95.

Both models use information about the 10 previous measurements to predict 4 steps (1 h) ahead utilizing the sliding window algorithm (see, e.g., [33]). This short-term prediction offers a balance between prediction accuracy and computational efficiency and allows for near real-time decision-making. Unlike ARIMA, which only allows the use of a single feature, the LSTM includes two additional features—the time and the type of day (weekday or weekend).

**Figure 4:**
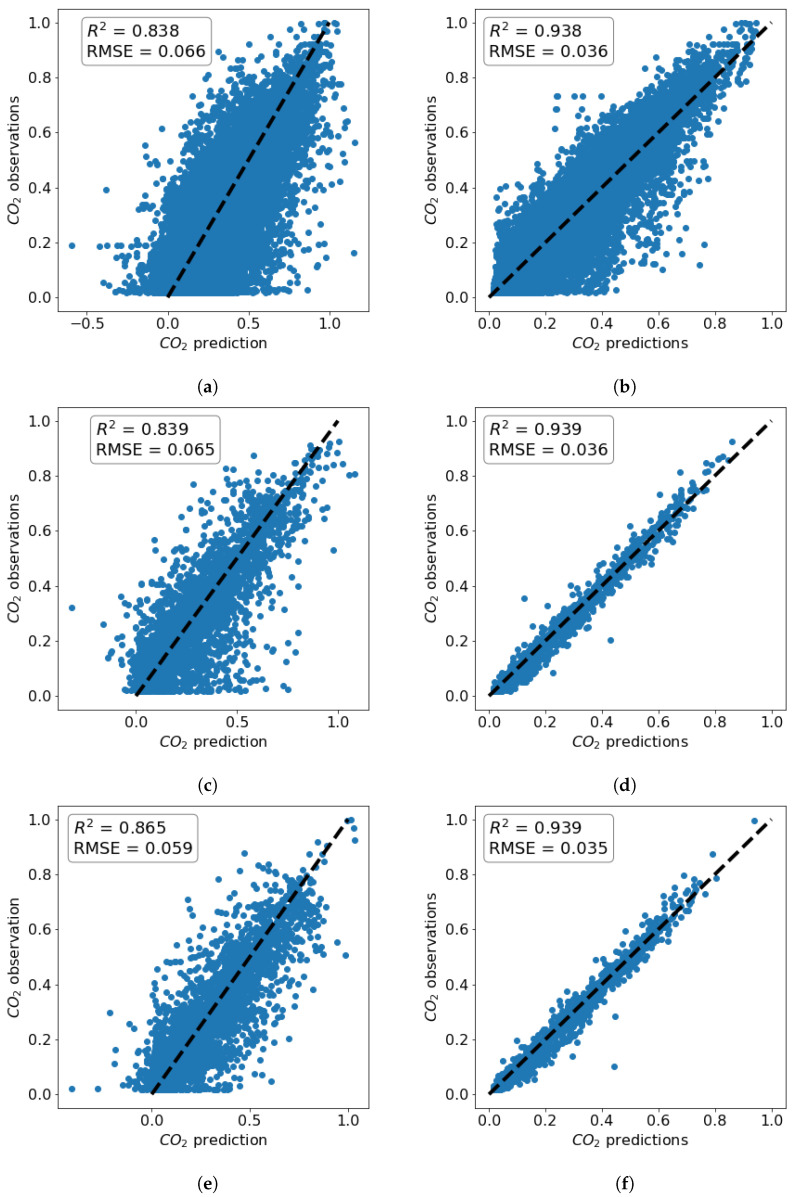
CO${}_{2}$ predictions vs. measurements plots (**a**) using ARIMA on the training set; (**b**) using LSTM on the training set; (**c**) using ARIMA on the validation set; (**d**) using LSTM on the validation set; (**e**) using ARIMA on the testing set; (**f**) using LSTM on the testing set.

Both models were run on a Dell Latitude 7400 with 4-core Intel(R) Core(TM) i7-8665U CPU and 16 GB of RAM, and their computational time was 45 min and 8 h in favor of the LSTM model. The results of the training process for the two models are shown in Figure 4. It can be seen that the coefficient of determination, $R^{2}$, in all cases is higher when LSTM is used. The results for temperature and relative humidity exhibit a qualitatively similar behavior to that of CO${}_{2}$, and are thus omitted here for brevity. As a result, LSTM is used in the further prediction of temperature, relative humidity, and overall comfort of the indoor conditions.

### 3.2. Comfort Conditions Prediction

**Figure 5:**
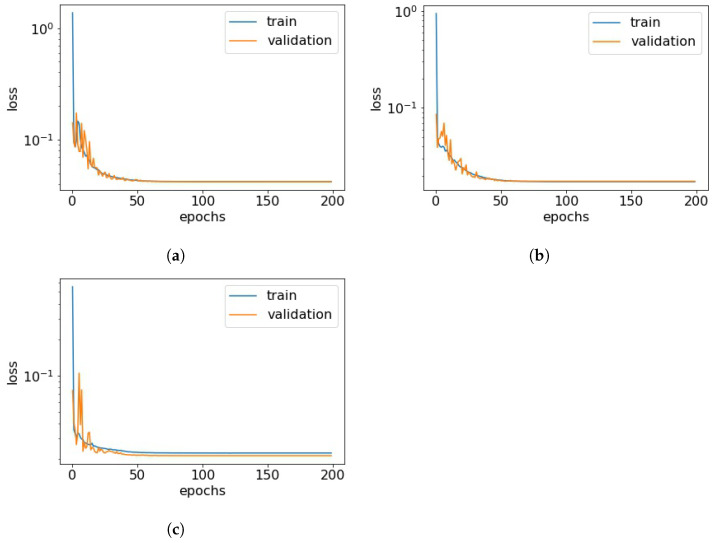
Loss function plots for (**a**) CO${}_{2}$, (**b**) temperature, and (**c**) relative humidity.

**Figure 6:**
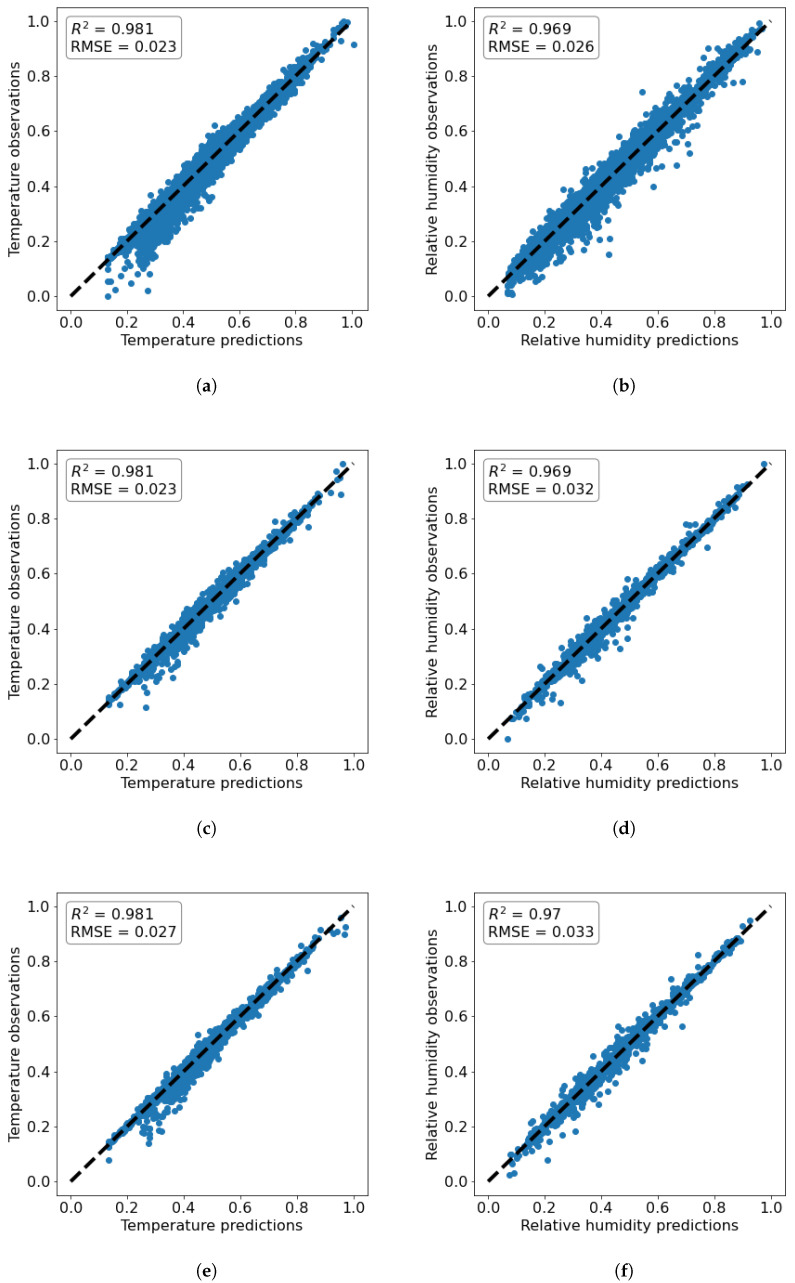
LSTM predictions vs. measurements plots (**a**) of temperature on the training set; (**b**) of relative humidity on the training set; (**c**) of temperature on the validation set; (**d**) of relative humidity on the validation set; (**e**) of temperature on the testing set; (**f**) of relative humidity on the validation set.

**Figure 7:**
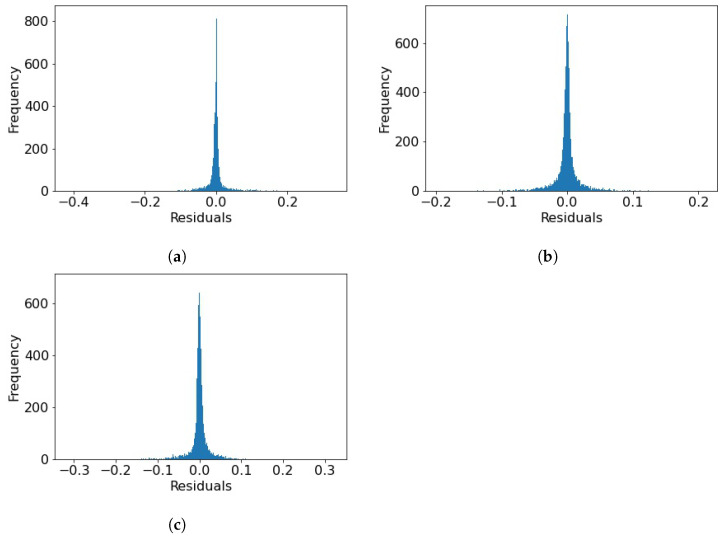
Residuals plots for the prediction of (**a**) CO${}_{2}$, (**b**) temperature, and (**c**) relative humidity.

Initially, the comfort conditions in the kindergarten room are determined based on the measurements and Table 3 so that every recording is assigned a category between 1 and 27. CO${}_{2}$ concentrations, temperature, and relative humidity are then all predicted 1 hour ahead using three separate LSTM models utilizing the architecture described in Section 3.1 and the loss functions of the training processes can be seen in Figure 5. This approach allows for a detailed understanding of the predicted values for each parameter and the factors influencing the comfort conditions. The results are presented in Figure 6. The coefficients of determination for temperature and relative humidity show a very high prediction accuracy. In addition, Figure 7 presents residual plots for all three predicted variables. The distribution of residuals suggests that the models are performing well, and the cluster around zero implies that the predicted values are close to the actual values. However, in all cases outliers can also be observed, which signals that the models could still be improved, and further investigation to identify the factors that contribute to those deviations may be needed in future work. Based on the predictions for these three variables, a forecast for the comfort conditions is also formed. A report for a post-analysis classification is presented in Table 5, which demonstrates the high precision in discovering the correct comfort categories. It can be seen that the comfort conditions are forecast correctly with an average accuracy of 91%, which indicates a good ability of the model to correctly identify instances across all categories. The zero precision of the obvious outliers in Categories 14 and 15 can potentially be attributed to the lack of sufficient data (a single point for each) in those categories, despite the apparent lack of correlation between the size of the support and precision counts.

**Figure 8:**
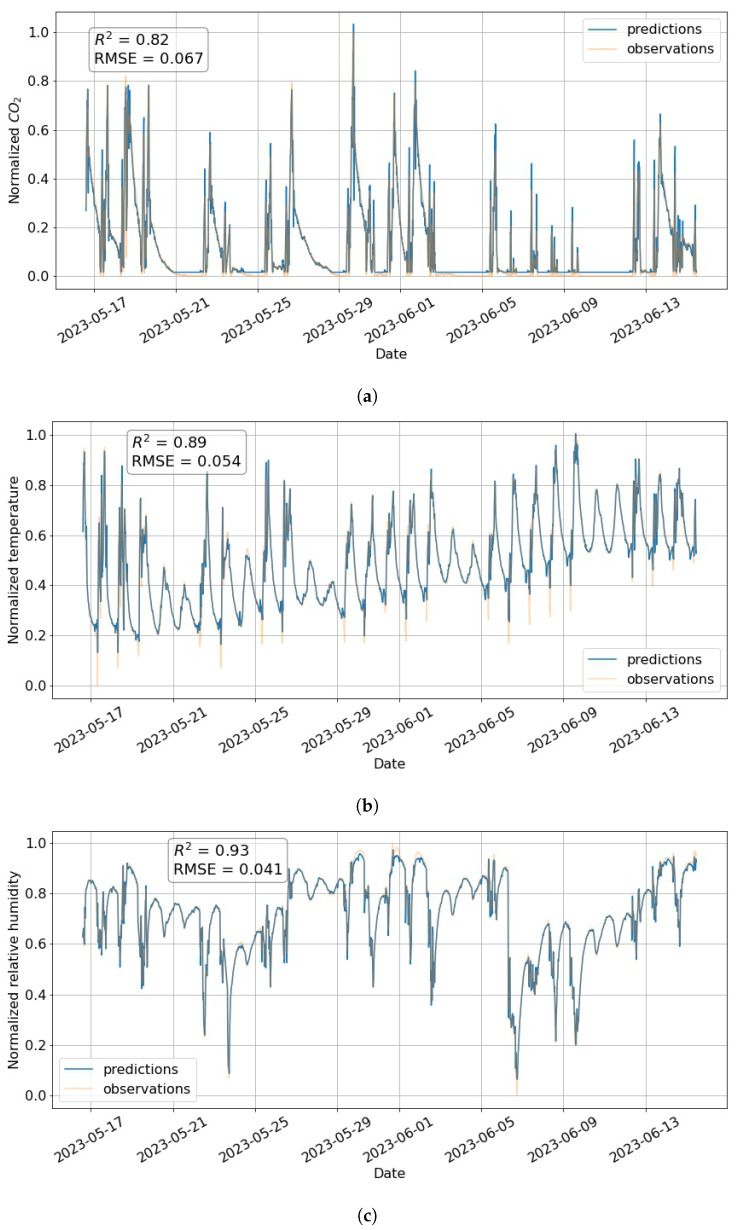
(**a**) CO${}_{2}$, (**b**) temperature, and (**c**) relative humidity predictions vs. measurements plots obtained using LSTM on the 30-day testing data set in 2023.

To put those figures in a more applied perspective, a time domain comparison is presented in Figure 8 showing the results on the additional data set covering a period of 30 days in 2023. Despite the good accuracy, a decrease in the coefficient of determination is notable for all three variables. The forecast of CO${}_{2}$ concentrations exhibits the largest deterioration, which can most clearly be seen in the prediction of the baseline and some of the peaks. This indicates the need for periodic retraining to ensure sustained accuracy in real-time predictions.

## 4. Conclusions

This study highlights the importance of monitoring and ensuring healthy indoor environments, particularly in educational facilities such as kindergartens. Poor indoor conditions can have significant implications for the well-being and learning efficiency of children. The research employed real-time monitoring and ML techniques to predict environmental conditions. The results demonstrate that the LSTM model outperforms a classical AR model in predicting CO${}_{2}$ concentrations, providing higher accuracy and shorter computational time. Furthermore, the study addresses the prediction of comfort conditions based on temperature, relative humidity, and CO${}_{2}$ levels. The forecast comfort conditions align closely with the actual conditions, achieving an average accuracy of 91%, when using the LSTM model.

These findings emphasize the significance of proactive measures to mitigate indoor air quality issues in educational settings. Implementing effective ventilation strategies, maintaining optimal temperature and humidity levels, and addressing CO${}_{2}$ buildup are essential for creating a healthy and conducive learning environment for children. The integration of ML techniques, as demonstrated in this study, can serve as valuable tools for continuous monitoring and prediction of indoor conditions, facilitating timely interventions and promoting the well-being of occupants.

Further research will focus on refining and expanding the predictive models to include additional factors that influence indoor air quality, examine different sliding window horizons, and study the effect of dependence between temperature, humidity, and CO${}_{2}$, which were considered independent in this study. Moreover, investigation into PM${}_{2.5}$ variations, especially with additional threshold levels, could also be a subject of future research in contexts where its levels are more consistently elevated. Additionally, the results obtained in this study can be used as the foundation of a computational fluid dynamics (CFD) simulation, which would offer a better understanding of the airflow patterns. This would enable the evaluation of different ventilation strategies, such as natural ventilation, mechanical ventilation, or a combination of both, to determine their effectiveness in maintaining a healthy indoor environment.

## Figures and Tables

**Table 1:** Descriptive statistics of the cleaned data set.

	T (${}^{{}^{\circ}}$C)	RH (%)	CO${}_{2}$ (ppm)	PM${}_{2.5}$ (μg/m${}^{3}$)
mean	21.14	41.96	753.8	7.96
std	3.14	7.25	436.4	4.59
min	12.93	19.47	374.0	0.00
25%	18.48	36.87	439.0	4.00
50%	20.55	41.73	568.3	7.00
75%	23.60	46.70	904.8	10.67
max	31.04	66.70	3247.0	38.00

**Table 2:** Threshold values for indoor comfort conditions.

Parameter	L1	L2
Temperature (${}^{{}^{\circ}}$C)	18	21
Relative Humidity (%)	30	60
Carbon Dioxide (ppm)	600	1000

**Table 3:** Indoor comfort conditions categorization.

N.	Temperature, T (${}^{{}^{\circ}}$C)	Relative Humidity, RH (%)	Carbon Dioxide, CO${}_{2}$ (ppm)
27	T above L2	RH above L2	CO${}_{2}$ above L2
26	T above L2	RH below L1	CO${}_{2}$ above L2
25	T below L1	RH above L2	CO${}_{2}$ above L2
24	T above L2	RH between L1 and L2	CO${}_{2}$ above L2
23	T below L1	RH below L1	CO${}_{2}$ above L2
22	T below L1	RH between L1 and L2	CO${}_{2}$ above L2
21	T between L1 and L2	RH above L2	CO${}_{2}$ above L2
20	T between L1 and L2	RH below L1	CO${}_{2}$ above L2
19	T between L1 and L2	RH between L1 and L2	CO${}_{2}$ above L2
18	T above L2	RH above L2	CO${}_{2}$ between L1 and L2
17	T above L2	RH below L1	CO${}_{2}$ between L1 and L2
16	T above L2	RH between L1 and L2	CO${}_{2}$ between L1 and L2
15	T below L1	RH above L2	CO${}_{2}$ between L1 and L2
14	T between L1 and L2	RH above L2	CO${}_{2}$ between L1 and L2
13	T above L2	RH above L2	CO${}_{2}$ below L1
12	T above L2	RH below L1	CO${}_{2}$ below L1
11	T above L2	RH between L1 and L2	CO${}_{2}$ below L1
10	T below L1	RH above L2	CO${}_{2}$ below L1
9	T below L1	RH below L1	CO${}_{2}$ between L1 and L2
8	T between L1 and L2	RH above L2	CO${}_{2}$ below L1
7	T below L1	RH between L1 and L2	CO${}_{2}$$CO_{2}$ between L1 and L2
6	T between L1 and L2	RH between L1 and L2	CO${}_{2}$ between L1 and L2
5	T below L1	RH below L1	CO${}_{2}$ below L1
4	T between L1 and L2	RH below L1	CO${}_{2}$ between L1 and L2
3	T below L1	RH between L1 and L2	CO${}_{2}$ below L1
2	T between L1 and L2	RH below L1	CO${}_{2}$ below L1
1	T between L1 and L2	RH between L1 and L2	CO${}_{2}$ below L1

**Table 4:** RNN architecture. Shape format reads (number of samples propagated through network, number of neurons in layer) or (number of samples propagated through network, number of time steps, number of neurons in layer). None denotes determination at runtime.

Layer	Type	Shape	Parameters
1	LSTM	(None, 10, 64)	17,408
2	LSTM	(None, 64)	33,024
3	Dense	(None, 32)	2080
4	Dense	(None, 4)	132

**Table 5:** Comfort conditions classification report. ‘Precision’ is the ratio of correct positive predictions to all positive predictions; ‘recall’ is the ratio of correct positive predictions to all positive instances; ‘f1-score’ is the weighted harmonic mean between precision and recall; ‘support’ indicates the number of occurrences of each class in the data set.

N.	Precision	Recall	f1-Score	Support
1	0.92	0.94	0.93	5098
2	0.75	0.79	0.77	317
3	0.92	0.93	0.93	2406
4	0.63	0.64	0.63	233
5	0.89	0.54	0.67	78
6	0.90	0.87	0.89	3571
7	0.91	0.93	0.92	1896
8	0.67	0.50	0.57	4
9	0.31	0.19	0.24	21
11	0.98	0.96	0.97	8524
12	0.88	0.73	0.80	173
13	0.96	0.90	0.93	219
14	0.00	0.00	0.00	1
15	0.00	0.00	0.00	1
16	0.81	0.84	0.82	2090
17	0.70	0.74	0.72	245
18	0.61	0.45	0.52	31
19	0.91	0.89	0.90	2861
20	0.58	0.53	0.55	62
21	0.94	0.94	0.94	17
22	0.89	0.91	0.90	570
24	0.87	0.92	0.90	2865
26	0.75	0.82	0.78	211
27	0.96	0.80	0.87	30
accuracy			0.91	31,524
average	0.74	0.70	0.71	31,524
weighted avg	0.91	0.91	0.91	31,524

## Data Availability

The complete data presented in this study are available on request from the corresponding author.
